# A 41-gene signature derived from breast cancer stem cells as a predictor of survival

**DOI:** 10.1186/1756-9966-33-49

**Published:** 2014-06-06

**Authors:** Zhi-Qiang Yin, Jian-Jun Liu, Ying-Chun Xu, Jian Yu, Guo-Hui Ding, Feng Yang, Lei Tang, Bao-Hong Liu, Yue Ma, Yu-Wei Xia, Xiao-Lin Lin, Hong-Xia Wang

**Affiliations:** 1 Shanghai Renji Hospital, Shanghai Jiaotong University School of Medicine, Shanghai 200127, China; 2 Shanghai Center for Bioinformation Technology, Shanghai 201203, China; 3 Department of Oncology, Renji Hospital, School of Medicine, Shanghai Jiaotong University, Shanghai 200127, China

**Keywords:** Prognostic signature, Breast cancer, Stem cell

## Abstract

**Purpose:**

The aim of this study was to evaluate the ability of a 41-gene signature derived from breast cancer stem cells (BCSCs) to estimate the risk of metastasis and survival in breast cancer patients.

**Methods:**

The centroid expression of the 41-gene signature derived from BCSCs was applied as the threshold to classify patients into two separate groups—patients with high expression (high-EL) of the prognostic signature and patients with low expression (low-EL). The predictive ability of the 41-gene signature was evaluated by Cox regression model and was compared against other popular tests, such as Oncotype and MammaPrint.

**Results:**

Our results showed that the 41-gene prognostic signature was significantly associated with age (*P* = .0351) and ER status (*P* = .0095). The analysis indicated that patients in the high-EL group had a worse prognosis than those in the low-EL group in terms of both overall survival (OS: HR, 2.05, *P* = .009) and distant metastasis-free survival (DMFS: HR, 2.24, *P* = .002). Additionally, the 41-gene signature was an independent risk factor and separates patients based on estrogen receptor status. While comparable to Oncotype, the analysis demonstrated that the 41-gene signature had a better prognostic value in predicting DMFS and OS than AOL, NPI, St. Gallen, Veridex, and MammaPrint.

**Conclusions:**

This study confirms the utility of the 41-gene signature and adds to the growing evidence that gene expression signatures of BCSCs have clinical potential to predict patient outcome and aid in treatment choice.

## Introduction

Personalized medicine, the selection of therapy based on a patient’s individual characteristics, may result in better outcomes than the use of generalized medicine [1-4]. Prognostic factors commonly applied in breast cancer include age, tumor size, lymph node involvement, pathological grade, and status of HER-2, Ki-67, and several hormone receptors, including both estrogen receptor (ER) and progesterone receptor (PR) [5,6]. Although several guidelines have been developed to assist clinicians in selecting patients who are at high risk of recurrence, it still remains a challenge to distinguish patients who have poor prognosis and require demanding adjuvant systemic therapy from those who could be spared such treatment. Due to the complexity of the disease, several other factors have been investigated for their potential to predict breast cancer outcome. However, most have only limited predictive power [7,8].

Recent findings support the concept that a rare population of cells, termed cancer stem-like cells (CSCs), is the cellular origin of cancer [9,10]. Such findings imply that it is these CSCs that are responsible for tumor initiation, progression, and response to therapy [11,12]. Therefore, an advance in our knowledge of the properties of CSCs has become a topic of considerable interest.

We previously identified a rare population of breast cancer stem cells (BCSCs) from tissue [13,14]. Human cancer is characterized by high heterogeneity in gene expression and phenotype, both of which influence tumor growth rate and drug sensitivity*.* We performed expression profiling to identify signaling pathways enriched in BCSCs. According to the gene expression profile, we found that sixty-three probe sets corresponding to forty-one genes showed greater than a four-fold difference in BCSCs compared to non-BCSCs. We hypothesized that this BCSC signature might be useful as a classification system since it outperformed most other clinical variables in predicting the likelihood of distant metastases and overall survival (OS) in breast cancer patients.

A more accurate means of prognostication in breast cancer will improve the selection of patients for adjuvant systemic therapy and will improve clinical decisions and strategies used to treat patients with this disease. Therefore, the present study was conducted to further evaluate the forty-one gene signature as a tool to accurately estimate the risks of metastases and survival in breast cancer patients.

## Methods

### Database of patients

Normalized gene expression data, together with the patient’s characteristics, were retrieved from the public GEO database (http://www.ncbi.nlm.nih.gov/geo; accession number GSE7390). For each patient, the information generated from the dataset included surgery type, angioinvasion (lymph vascular invasion), histopathological grading, ER status, OS, distant metastasis-free survival (DMFS), clinical risk group according to St. Gallen criteria, National Provider Identifier (NPI) criteria, Adjuvant online (AOL) (http://www.adjuvantonline.com), Veridex signature, MammaPrint, and Oncotype Dx.

### Study design

The 41 DEGs (differential expressed genes) correspond to 63 probe sets. Based on these probe sets, we obtained relevant expression values of patients from GSE7390. The centroid expression of these probe sets was applied as the patient classification threshold. Based on the threshold of the prognostic signatures, breast cancer patients in the dataset can be classified into two separate groups—patients with high expression (high-EL) of the prognostic signature and patients with low expression (low-EL) of the prognostic signature.

### Statistical analysis

To assess the prognostic value of the 41-gene signature, we utilized the Kaplan-Meier estimator to plot survival curves and the log-rank test to compare differences between two groups [15]. Fisher's exact test was employed to investigate the relevance between the 41-gene signature and clinical factors. Standard Cox proportional hazards regression were implemented to predict OS and DMFS. The performance of the 41-gene signature and other standard criteria, including AOL, NPI, St. Gallen, Veridex, Oncotype DX, and MammaPrint were evaluated in terms of LHR and Akaike information criterion (AIC) in a full model (all systems included) and in a series of reduced models where each interested factor was removed once each time. When removed from the full model, the best option results in the largest drop in LHR χ^2^ and an increase in AIC. All statistical analyses were performed by the R programming package with rms.

End points considered in this study were time from diagnosis to distant metastases (DMFS) and OS, which was defined as time from diagnosis to death by any cause. The linearity of the relation between the relative hazard ratio and the diameter of the tumor, age, and ER expression level were tested using the Wald test for nonlinear components of restricted cubic splines. No evidence for nonlinearity was found (*P* = .83 for age, *P* = .75 for tumor diameter, *P* = .65 for the number of positive nodes, and *P* = .27 for ER expression). We evaluated whether the hazard ratio was proportional using the method of Grambsch and Therneau.

## Results

### Characteristics of patients

The study was carried out with frozen archived tumor material from early stage breast cancer patients using the Affymetrix HG-U133A chip as previously described by the TRANSBIG consortium [16].

### Pattern of the 41-gene expression profile in breast cancer patients

Functional annotation of these 41 genes (Table 1) provides insight into the underlying biological mechanism leading to breast cancer tumorigenesis and the cellular signaling pathways regulating BCSCs.

**Table 1:** List and functional annotation of the 41 genes in the study

**ID**	**Gene name**	**Function**
23586	DDX58	DEAD box protein.
1041	CDSN	Corneocdesmosin, is a secreted protein found in corneodesmosomes.
259230	SGMS1	Sphingomyelin synthase 1.
81669	LOC643556	Similar to Aurora kinase A-interacting protein (AURKA-interacting protein).
54809	SAMD9	A sterile alpha motif domain-containing protein, regulating cell proliferation/apoptosis.
6352	CCL5	Chemokine (C-C motif) ligand 5.
90362	FAM110B	Family with sequence similarity 110, member B.
4176	MCM7	DNA replication licensing factor, Minichromosome maintenance complex component 7.
4938	OAS1	Encodes a member of the 2-5A synthetase family, essential proteins involved in the innate immune response to viral infection.
4939	OAS2	A member of the 2-5A synthetase family.
27289	RND1	A small (~21 kDa) signaling G protein, and is a member of the Rho family of GTPases.
3909	LAMA3	Laminin, alpha 3.
10268	RAMP3	Receptor (G protein-coupled) activity modifying protein 3.
5514	PPP1R10	A protein with similarity to a rat protein that has an inhibitory effect on protein phosphatase-1.
6324	SCN1B	Sodium channel, voltage-gated, type I, beta.
9687	GREB1	An estrogen-responsive gene.
11151	CHRO1A	Coronin, actin binding protein, 1A.
3434	IFIT1	Interferon-induced protein with tetratricopeptide repeats 1.
3433	IFIT2	Interferon-induced protein with tetratricopeptide repeats 2.
3437	IFIT3	Interferon-induced protein with tetratricopeptide repeats 3.
634	CEACAM1	Carcinoembryonic antigen-related cell adhesion molecule 1 (biliary glycoprotein).
4680	CEACAM6	Carcinoembryonic antigen-related cell adhesion molecule 6.
79971	WLS	wntless homolog (Drosophila).
3456	IFNB1	Interferon, beta 1, fibroblast.
9442	MED27	Mediator complex subunit 27, the activation of gene transcription.
8638	OASL	2′-5′-oligoadenylate synthetase-like gene.
1316	KLF6	A member of the Kruppel-like family of transcription factors.
55422	ZNF331	A zinc finger protein containing a KRAB (Kruppel-associated box) domain.
3853	KRT6A	A member of the keratin gene family.
653	BMP5	A member of the bone morphogenetic protein family.
10916	MAGED2	Melanoma-associated antigen D2.
3627	CXCL10	A chemokine of the CXC subfamily and ligand for the receptor CXCR3.
3433	IHIH2	Interferon induced with helicase C domain 2.
3569	IL6	Interleukin 6.
3576	IL8	Interleukin 8.
347733	TUBB2B	A beta isoform of tubulin, which binds GTP and is a major component of microtubules.
629	CFB	Complement factor B.
56999	ADAMTS9	A disintegrin and metalloproteinase with thrombospondin motifs protein family.
6482	HS.374257	ST3 beta-galactoside alpha-2,3-sialyltransferase 1.
90627	STARD13	StAR-related lipid transfer (START) domain containing 13.
64135	IFIH1	Interferon induced with helicase C domain 1.

**Figure 1:**
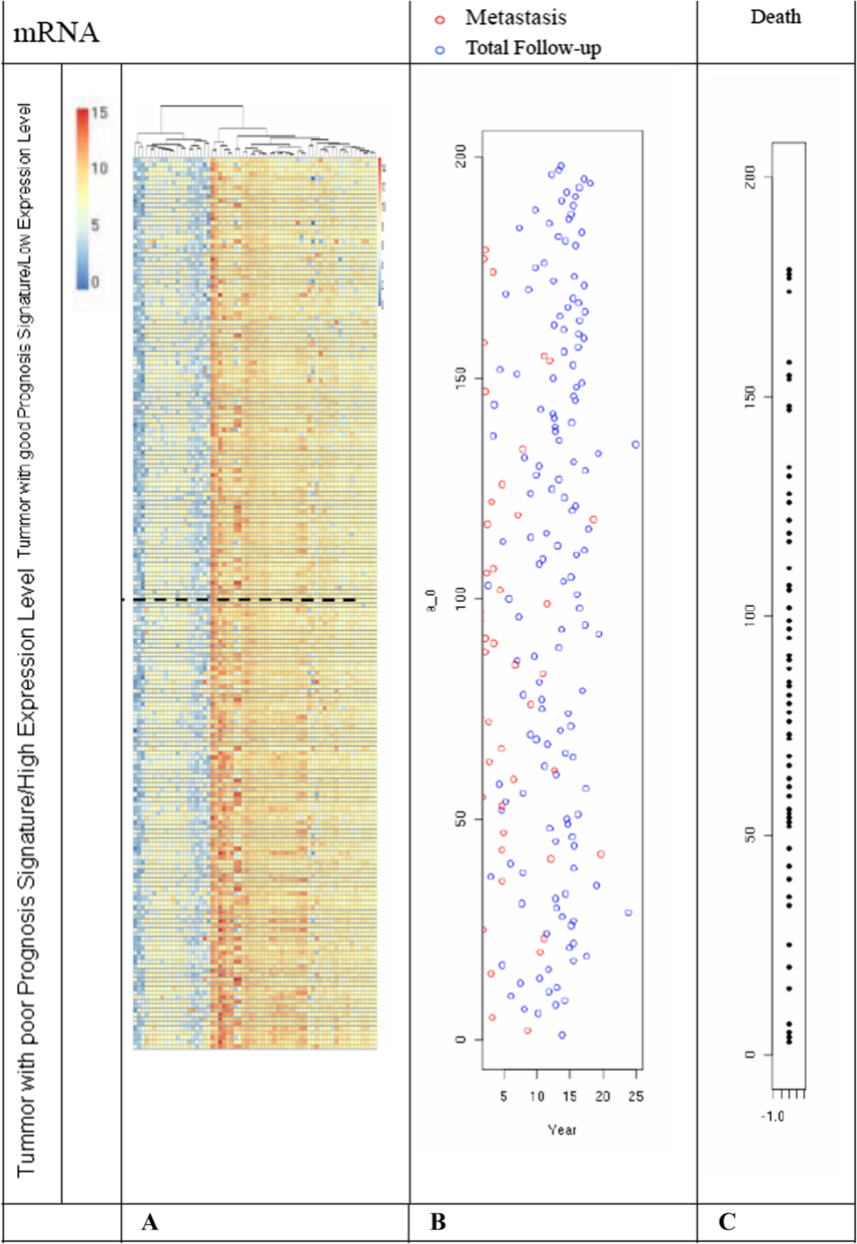
**Pattern of expression of genes used to determine the prognosis and clinical characteristics of 198 breast cancer patients.** Panel **A** shows the pattern of expression of the 63 marker genes in a series of 198 consecutive patients with breast carcinoma. Each row represents the prognostic profile of the 63 marker genes for one tumor, and each column represents the relative level of expression of one gene. The tumors are numbered from 1 to 198 on the y axis, and the genes are numbered from 1 to 63 on the x axis. Panel **B** shows the time in years to distant metastasis as a first event for those in whom this occurred, and the total duration of follow-up for all other patients. Panel **C** shows the living status. The black dots represent the number of patients who died.

The gene-expression values of the 41 markers for all 198 tumors in this study are shown in Figure 1. As shown in Figure 1A, red indicates increased mRNA expression in the tumor compared to the reference; green indicates low level expression. The dotted line represents the previously determined threshold between a good-prognosis signature and a poor-prognosis signature. Tumors are rank-ordered according to the expression level of the 41 prognostic genes in tumors from 198 patients. Figure 1B shows the time in years to distant metastasis as a first event of this occurrence, as well as the total duration of follow-up for all patients. Figure 1C shows the living status of these breast cancer patients.

### Association between the 41-gene prognostic signature and clinical variables

The 198 patients were divided into two groups based on high expression level (high-EL, n = 99) and low expression level (low-EL, n = 99), similar to earlier reports [17]. These levels correspond to a poor prognostic signature and a good prognostic signature, respectively. To gain insight into the relationship between the 41-gene prognostic signature and clinical variables, we performed correlation analysis with histopathologic data of patients, such as, age, surgery type, grade, and ER expression as determined by immunohistochemical (IHC) staining. The results showed that the 41-gene prognostic signature was significantly associated with age (*P* = .0351) and ER status (*P* = .0095). Patients in the high-EL group were younger in age and had ER-negative tumors. There was also a slightly significant association with tumor grade. However, the p value showed no statistical significance.

### Analysis of DMFS and OS based on the prognostic signature

Our analysis indicated that the likelihood of patients developing distant metastasis at 5 years and 10 years was higher in the low-EL group than in the high-EL group (5 year DMFS: 88% versus 75%, respectively; 10 years DMFS: 83% versus 64%, respectively). Prolonged OS was also observed in low-EL patients.

Additionally, multivariate analysis was conducted to adjust for confounding variables including age, tumor size, tumor grade, and ER status. Results confirmed that the 41-gene signature was an independent prognostic factor for these breast cancer patients (OS: HR, 1.96, *P* = .02; DMFS: HR, 2.09, *P* = .008).

### Survival comparison between the new markers and other standard criteria

**Figure 2:**
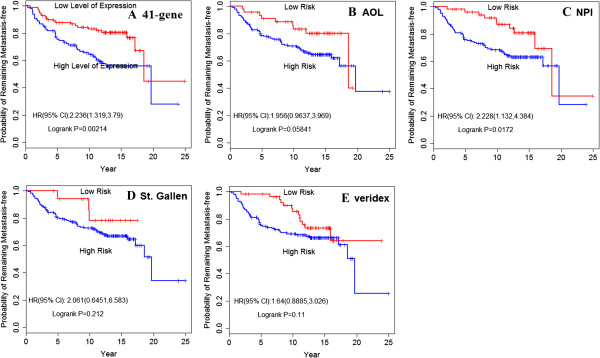
**Kaplan-Meier analysis of the probability that patients would remain free of distant metastasis among all patients. A**. prediction value of DMFS by the 41-gene signature. Patients were divided into those with a good-prognostic signature and those with a poor prognostic signature according to gene-expression profiling; **B**. prediction value of OS by AOL consensus criteria; **C**. prediction value of DMFS by NPI consensus criteria; **D**. prediction value of DMFS by St. Gallen criteria; **E**. prediction of Veridex signature. The p values were calculated by log-rank test.

**Figure 3:**
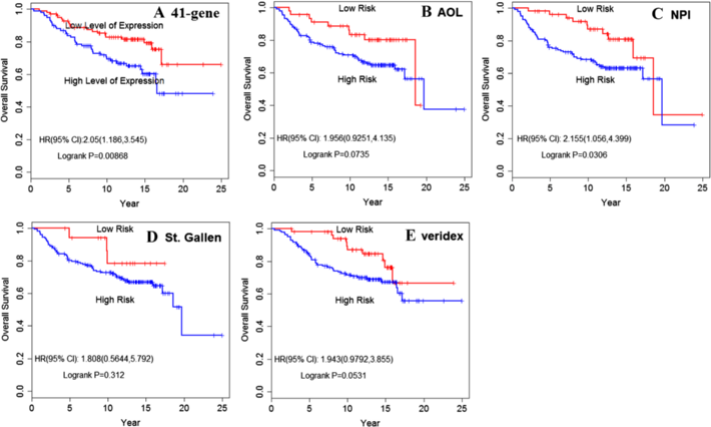
**Kaplan-Meier analysis of the probability of OS. A**. prediction value of OS by the 41-gene signature. Patients were divided into those with a good-prognostic signature and those with a poor prognostic signature according to gene-expression profiling; **B**. prediction value of OS by AOL consensus criteria; **C**. prediction value of OS by NPI consensus criteria; **D**. prediction value of OS by St. Gallen criteria; **E**. Prediction value of Veridex signature. The p values were calculated by log-rank test.

The Kaplan-Meier curve (Figure 2A) showed a significant difference (HR, 2.236; 95% confidence interval [CI], 1.319 to 3.79) in the probability that patients would remain metastasis-free in the low-EL compared to the high-EL group (*P* = .002). The 41-gene prognostic signature was also extremely useful in predicting the outcome of OS (HR, 2.050; 95% CI, 1.186 to 3.545; *P* = 0.009) (Figure 3A).

To obtain a more powerful estimate of the signature in predicting clinical outcome, we compared the 41-gene prognostic signature with other commonly used criteria, such as AOL, NPI, St. Gallen, and Veridex. Based on this analysis, patients in the database can be divided into a high-risk group and a low-risk group according to various histologic and clinical characteristics. We calculated DMFS and OS according to these different prognostic profiles. The analysis indicated that the 41-gene signature had the best prognostic value in predicting DMFS (*P* = .058 for AOL; *P* = 0.017 for NPI; *P* = .11 for Veridex; and *P* = .212 for St. Gallen) (Figure 2B, 2C, 2D, 2E) and OS (*P* = .074 for AOL; *P* = .031 for NPI; *P* = .053 for Veridex; and *P* = .312 for St. Gallen) for early breast cancer patients (Figure 3B, 3C, 3D, 3E).

### Prognostic value in high-risk patients defined by other standard criteria

**Figure 4:**
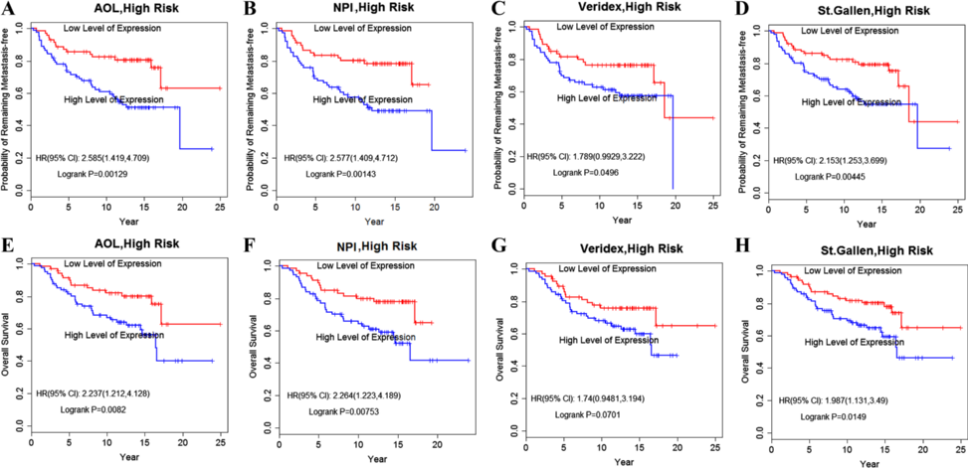
**The 41-gene signature was a stronger predictor of clinical outcome. A**. prediction value of DMFS in high-risk patients defined by AOL criteria; **B**. prediction value of DMFS in high-risk patients defined by NPI criteria; **C**. prediction value of DMFS in high-risk patients defined by Veridex criteria; **D**. prediction value of DMFS in high-risk patients defined by St. Gallen criteria; **E**. prediction value of OS in high-risk patients defined by AOL criteria; **F**. prediction value of OS in high-risk patients defined by NPI criteria; **G**. prediction value of OS in high-risk patients defined by Veridex criteria; **H**. prediction value of OS in high-risk patients defined by St. Gallen criteria.

**Figure 5:**
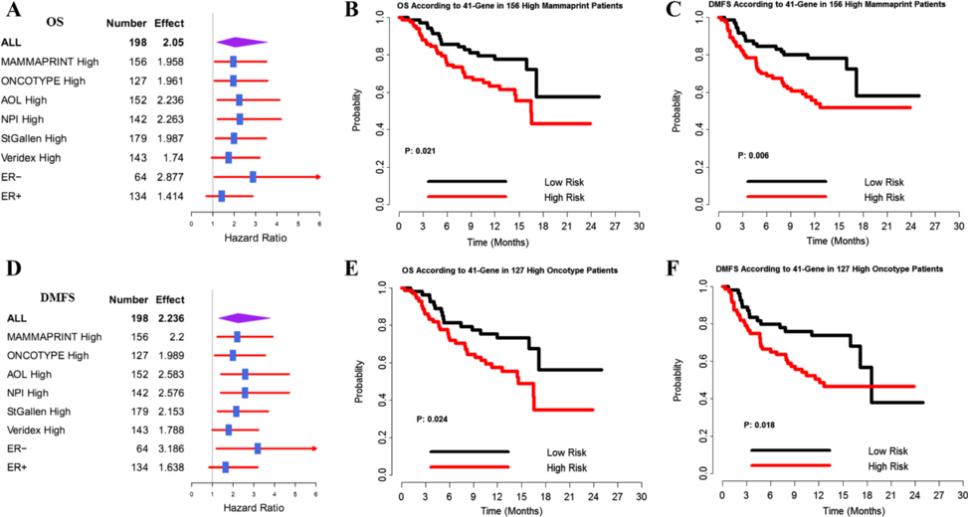
**Prognostic value represented by forest plot in patients defined by other standard criteria. A**. prognostic value of OS represented by forest plot; **B**. prognostic value of OS in high-risk patients defined by Mammaprint; **C**. prognostic value of DMFS in high-risk patients defined by Mammaprint; **D**. prognostic value of DMFS represented by forest plot; **E**. prognostic value of OS in high-risk patients defined by Oncotype; **F**. prognostic value of DMFS in high-risk patients defined by Oncotype.

The 41-gene prognostic signature was also highly predictive of the risk of DMFS and OS among the subgroup of patients, which were thought to be high risk according to other existing criteria. As shown in the Kaplan-Meier curves, we found significant differences in the probability of remaining metastasis-free between the high-EL signature and the low-EL signature, even though all were assigned to the high-risk group based on other criteria (*P* = .001 for AOL; *P* = .001 for NPI; *P* = .049 for Veridex; *P* = .004 for St. Gallen; *P* = .006 for MammaPrint; and *P* = .018 for Oncotype Dx) (Figure 4A, 4B, 4C, 4D; Figure 5C, 5F). A similar trend was observed when assessing OS (Figure 4E, 4F, 4G, 4H; Figure 5B, 5E). Thus, the new prognostic signature more accurately predicts breast cancer survival rate (or metastasis) than other histologic and clinical characteristics. These results highlight the value of the prognosis profile and the robustness of the profiling technique.

### Comparison of the prognostic value of the 41-gene signature with Oncotype Dx and MammaPrint

To assess the concordance of the 41-gene signature with published prognostic gene signatures, we implemented the original algorithms of the Oncotype Dx (Genomic Health) and MammaPrint (Agendia) gene signatures and applied them to the 41-gene signature in our compendium of microarray datasets.

Using data from the 198 patients with node-negative tumors, we analyzed the prognostic value of the 41-gene signature, Oncotype Dx, MammaPrint, and other criteria (Table 2). The results of multivariate analysis indicated that there was significant prognostic power for the 41-gene signature (*P* = .03) and Oncotype Dx (*P* = .002). However, there was no statistically significant difference observed for the analysis using MammaPrint (*P* = .647), AOL criteria (*P* = .551), NPI criteria (*P* = .16), St. Gallen criteria (*P* = .383), or Veridex criteria (*P* = .335).

**Table 2:** Comparison of the prognostic value of 41-gene signature with other risk assessment criteria

	**OS**			**DMFS**		
	**ΔLHR**	**P-value**	**ΔAIC**	**ΔLHR**	**P-value**	**ΔAIC**
Signature	−4.929	0.03*	2.929	−10.513	0.008*	5.475
ONCOTYPE	−13.286	0.002*	11.286	−13.734	0.004*	8.696
MAMMAPRINT	−0.221	0.647	−1.779	−3.038	0.986	−2
AOL	−0.377	0.551	−1.623	−3.325	0.601	−1.713
NPI	−3.658	0.16	2.764	−6.823	0.131	2.756
St. Gallen	−0.724	0.383	−1.276	−3.33	0.582	−1.708
Veridex	−0.991	0.335	−1.009	−3.987	0.343	−1.051

The independent contribution of each interested factor to patient outcome was assessed by first removing the factors concerned and then calculating the difference of LHR and AIC. A larger drop of LHR and an increase in AIC indicate a higher significance of the removed system.

ΔLHR, change of likelihood ratio between full model fitting and one concerned system removed; ΔAIC, change of Akaike information criterion between full model fitting and one concerned system removed; *, statistical significant.

We further investigated the prognostic ability of the 41-gene signature under different definitions of “high risk” using forest plots. As shown in Figure 5A and Figure 5D, the new markers displayed good predictive ability in almost all subgroups except for ER-positive patients.

### Subgroup analysis according to ER status

**Figure 6:**
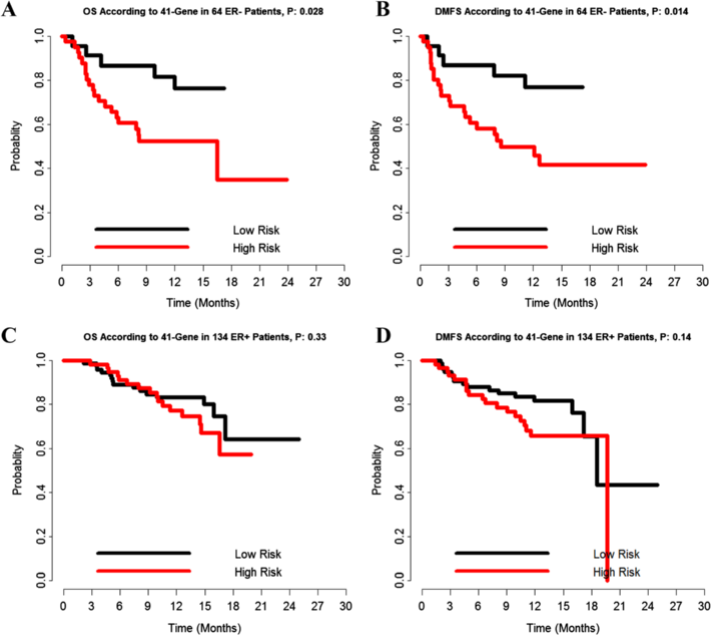
**The prognostic value of 41-gene signature in ER positive and ER negative patients. A**. prognostic value of OS in ER^−^ patients; **B**. prognostic value of DMFS in ER^−^ patients; **C**. prognostic value of OS in ER^+^ patients; **D**. prognostic value of DMFS in ER^+^ patients.

In order to discuss the impact of ER status on the 41-gene signature, we separately analyzed the predictive value of these markers in ER-positive and ER-negative patients. The survival curves were statistically significantly different between the high-EL patients and low-EL patients for DMFS (*P* = .014) and OS (*P* = .028) in ER negative patients, indicating a good predictive ability in this subgroup (Figure 6A, 6B). However, the signature did not show strong predictive ability for ER positive patients (Figure 6C, 6D). These curves confirmed earlier results from forest plot analysis (Figure 5A, 5D).

## Discussion

Previous studies linking gene expression profiles to clinical outcome in breast cancer have demonstrated that the potential for distant metastasis and OS probability may be attributable to biological characteristics of the primary tumor [18-21]. In their seminal work, Paik et al. [22] reported that a 21-gene recurrence score (RS) assay quantifies the likelihood of distant recurrence in women with ER-positive, lymph node-negative breast cancer treated with adjuvant tamoxifen; it also predicts the magnitude of chemotherapy benefit. Perou et al. [23] identified tumors with distinct patterns of gene expression termed “basal type” and “luminal type”, using complementary DNA (cDNA) microarray to analyze breast cancer tissues. These subgroups differ with respect to disease outcome in patients with locally advanced breast cancer. Generally, it is agreed that patients with poor prognostic features benefit most from adjuvant therapy.

We previously identified a gene expression profile of 41-gene markers that is associated with BCSCs. Since BCSCs are considered to be the root of metastasis, promote recurrence of the malignancy, and are resistant to traditional therapy [24-27], we tested this profile in a series of 198 consecutive patients who were diagnosed with early breast cancer. The results showed that the 41-gene profile performed best as a predictor of DMFS by classifying patients into high-EL and low-EL groups. The prognostic signature is also a strong predictor of OS in patients with lymph node negative disease in this cohort.

To our knowledge, this is the first attempt at using cancer stem cell related markers as a prognostic signature predicting the survival and recurrence of breast cancer patients. This finding is important since the presence of cancer stem cells is a strong predictor of poor survival and resistance to traditional therapy. This finding also sheds new light on the common biological processes relevant for predicting outcome in breast cancer.

Comparing Figure 2A and Figure 3A, we see a strong correlation between the good-prognostic signature and DMFS (*P* = .002). Similar results were observed in the analysis of OS (*P* = .0086). To obtain a more useful estimate of clinical outcome, we calculated the probability of patients who remained free of distant metastasis and OS according to the prognosis profile. For this analysis, our results indicated that the prognostic signature was highly predictive of the risk of distant metastasis. Prolonged OS was also observed in patients with low expression of the 41-gene signature compared to patients in the high-EL group. These results highlight the value of prognostic profiles and the robustness of the profiling technique.

For the purpose of comparison, we also analyzed well-established criteria currently used in the clinic predicting clinical outcomes for breast cancer patients, such as AOL, NPI, St. Gallen, and Veridex. Figure 2 and Figure 3 shows the Kaplan-Meier estimates of the probability that patients would remain free of distant metastasis and OS among the 198 patients with lymph-node-negative breast cancer. In these analysis, patients were classified either by the 41-gene-expression profile or by another commonly used criteria, such as AOL, NPI consensus criteria, St. Gallen criteria, or Veridex criteria. The results indicated that only the NPI consensus criteria (*P* = .0172) predicted a statistically significant survival outcome in this cohort. It is worth noting that no statistical significance was observed for AOL, NPI, or St. Gallen criteria in predicting clinical outcome for this cohort of breast cancer patients.

MammaPrint [28] and Oncotype Dx [29] are currently commercially available diagnostic tests that quantify the likelihood of disease recurrence in women with early-stage breast cancer. Within this cohort, the analysis revealed that the 41-gene signature and Oncotype Dx both had strong prognostic value in predicting DMFS and OS in this 198 patient group. However, there was no statistically significant difference observed for the analysis with MammaPrint.

High-risk patients identified by AOL, NPI, St. Gallen, or Veridex criteria tended to have a lower likelihood of DMFS and OS than those classified according to the 41-gene expression profiling. This result indicates that both sets of the currently used criteria “misclassified” a clinically significant number of patients. Indeed, the high-risk group, defined according to these criteria, might include a number of patients who actually had a good-prognostic signature with a possible good outcome. Since both these subgroups contain some “misclassified” patients (who can be better identified through the prognosis signature), these patients might be mistreated in current clinical practice.

Based on our analysis, we predict that the 41-gene signature profile significantly associates with clinical outcome in the entire patient cohort. Thus, we further evaluated the prognostic utility of these 41-genes in ER positive and ER negative patients, respectively. In the subgroup analysis, there was a significant association between the 41-gene signature and both OS and DMFS in ER-negative breast cancer patients. In contrast, the signature did not show strong predictive ability for ER positive patients.

The molecular mechanisms regulating BCSCs are distinct from the mechanisms governing differentiated tumor cells. Our data indicate that classification of patients into high-risk and low-risk subgroups on the basis of the 41-gene prognostic profile could prove to be a very useful means of guiding adjuvant therapy in patients with lymph-node-negative breast cancer. This approach should also improve the selection of patients who would benefit from adjuvant systemic treatment, reducing the rate of both over-treatment and under-treatment. Even though these results are encouraging, a larger scale prospective study is required to confirm these results.

## Conclusion

The 41-gene prognostic profile demonstrates prognostic significance with strong capability of predicting DMFS and OS in node-negative breast cancer patients. This 41-gene signature of BCSCs was even more strongly associated with clinical outcomes compared with other existing criteria, such as AOL, NPI, Veridex, St. Gallen, and MammaPrint.

## Competing interests

There are no competing interests among the authors.

## Authors’ contributions

YZQ, LJJ, XYC, FY, TL, XYW, LXL, MY collected clinical information. XYW, YJ, DGH and LBH performed the statistical analysis. YZQ, LJJ and WHX participated in the design of the study. XYC and YJ drafted the manuscript. WHX revised the manuscript. All authors read and approved the final manuscript.
